# Clinical, Laboratory Characteristics, and Treatment Outcomes of Histoplasmosis Among Patients Admitted to a Referral Tertiary Care Hospital in Bangladesh

**DOI:** 10.7759/cureus.50813

**Published:** 2023-12-19

**Authors:** SK Jakaria Been Sayeed, Md Mujibur Rahman, Md Moniruzzaman, AKM Humayon Kabir, Md Uzzwal Mallik, Md Rockyb Hasan, Mohammad Golam-ur-Rahman, Bikash Chandra Mondal, Mohammad Arman Hossain, Mehrin Rahman

**Affiliations:** 1 Medicine and Rheumatology, National Institute of Neurosciences & Hospital, Dhaka, BGD; 2 Internal Medicine, Popular Medical College & Hospital, Dhaka, BGD; 3 Medicine, Dhaka Medical College Hospital, Dhaka, BGD; 4 Internal Medicine, Texas Tech University Health Sciences Center, Amarillo, USA; 5 Respiratory Medicine, National Institute of Diseases of the Chest and Hospital, Dhaka, BGD; 6 Internal Medicine, 250 Bedded TB Hospital, Dhaka, BGD

**Keywords:** outcome, liposomal amphotericin b, treatment, clinical and laboratory characteristics, histoplasmosis

## Abstract

Background: Histoplasmosis is a systemic mycosis caused by *Histoplasma capsulatum* (*H. capsulatum*). Systemic involvement of histoplasmosis usually occurs in immune-compromised patients, patients with AIDS, or those taking immunosuppressive therapy. The present study aims to describe the clinical and laboratory characteristics and treatment outcome of histoplasmosis as the diagnosis is challenging and management protocol differs.

Method: This retrospective study was done using a data registry at the medicine department of Dhaka Medical College Hospital. Here, patients received the standard treatment of histoplasmosis. Here, patients received the standard treatment of histoplasmosis, and clinical outcome was assessed at 3 months following starting standard treatment.

Result: A total of nine patients were enrolled, six (66.7%) had systemic histoplasmosis. Three were poultry workers, and the most common comorbidity was diabetes 3 (33.3%). Fever 7 (77.7%), weight loss 6 (66.7%), hyperpigmentation 5 (55.5%), cough 4 (44.4%), oral ulceration 4 (44.4%), lymphadenopathy 4 (44.4%), and hypotension 3 (33.3%) were the most common clinical presentations. Seven (77.7%) out of nine patients were cured of histoplasmosis; however, one died before initiating antifungal medications and another one died due to a hypersensitivity reaction to liposomal amphotericin B.

Conclusion: For local histoplasmosis, oral itraconazole is an effective antifungal medication. However, in disseminated Histoplasmosis, liposomal amphotericin B followed by oral itraconazole is still one of the preferable and effective treatment options. Clinicians should be aware of hypersensitivity reactions of liposomal amphotericin B and its management before giving an infusion.

## Introduction

Histoplasmosis is a systemic mycosis caused by *Histoplasma capsulatum* (*H. capsulatum*). Usually, clinical manifestations occur in several forms such as asymptomatic, acute or chronic pulmonary, and disseminated infection. Disseminated histoplasmosis (DH) is characterized by hematogenous dissemination with reticuloendothelial organ involvement. The reactivation of latent infection or significant exposure is responsible for this phenomenon. Systemic involvement of histoplasmosis usually occurs in immune-compromised patients with Acquired Immune Deficiency Syndrome (AIDS) or patients on immunosuppressive therapy [1,2]. Tuberculosis and histoplasmosis are commonly present in AIDS as disseminated infections so it is necessary to rule out causative pathogens by rapid antigen detection, fungal stain, and culture; although detection techniques are limited in many countries [3]. Moreover, uncontrolled diabetes is another risk factor for *Histoplasma* infection as it makes a person susceptible to infection but is not well described for *Histoplasma* infection [4]. Patients with systemic involvement may present with overwhelming infection, shock, respiratory distress, hepatic and renal failure, obtundation, and coagulopathy with a mortality rate of up to 50% even after intravenous amphotericin B treatment [5]. Severe *Histoplasma* infection should be treated with amphotericin B followed by oral itraconazole, and methylprednisolone for acute respiratory distress syndrome [6]. In Bangladesh, recently a good number of disseminated histoplasmosis cases have been reported in both immune-deficient and immune-competent patients [7-9]. However, treatment outcome with liposomal amphotericin B or itraconazole is very limited in Bangladesh. Here, we describe nine cases of histoplasmosis involving different organs both in immune-compromised and immune-competent patients with their clinical and laboratory characteristics and treatment outcomes.

## Materials and methods

Study settings and populations

This was a retrospective study, conducted at the Department of Medicine, Dhaka Medical College Hospital, the largest referral hospital in Bangladesh. Admitted patients who were diagnosed with histoplasmosis were enrolled. Diagnosis of histoplasmosis was made by clinical practice guidelines for the management of patients with histoplasmosis, 2007 [6]. Data were collected from hospital records.

Inclusion criteria

Patients more than 18 years of age, diagnosed with histoplasmosis were included. The diagnosis was confirmed by biopsy followed by histopathology with a specific fungal stain (periodic acid-Schiff/methenamine silver) and the presence of *H. capsulatum* within the macrophage.

Diagnosis

The definite diagnosis of histoplasmosis is made by demonstrating fungi on histopathology, cytopathology, or cultures. After staining with methenamine silver or periodic acid-Schiff, ovoid, narrow-based budding yeast forms can be seen within tissues or engulfed by macrophages.

Treatment protocol

For, disseminated cases liposomal amphotericin B 5 mg/kg per day in 5% dextrose in aqua was given intravenously at the rate of 20 drops per minute for consecutive 7 days followed by oral itraconazole 200 mg 8 hourly for 3 days, thereafter 200 mg 12 hourly for 12 weeks. For localized cases, itraconazole 200 mg 8 hourly for consecutive 3 days, thereafter 200 mg 12 hourly for the next 12 weeks.

Outcome

The primary outcome was to see the resolution of signs and symptoms of histoplasma infection among patients who received antifungal treatment. The secondary outcome was to observe adverse drug reactions and mortality among them. Clinical outcomes were assessed at 3 months following treatment. Here, cured means improvement of clinical features and systemic symptoms.

Statistical analysis

The continuous variable was expressed with the number, mean, and standard deviation (SD), and range while categorical values were expressed in frequency and percentage.

Investigations

For, each patient’s baseline investigations like complete blood count, urine routine examination, serum creatinine, alanine aminotransferase (ALT), aspartate aminotransferase (AST), plasma glucose, hemoglobin A1C (HbA1C), serum electrolyte, chest X-ray, ultrasonogram of the whole abdomen along with special investigations like CT (computed tomography) scan of the chest, abdomen, anti-HIV antibody, venereal disease research laboratory (VDRL) test for syphilis, Mantoux test, sputum for acid-fast bacilli (AFB) stain with GeneXpert, bronchoscopy with broncho-alveolar lavage, Immuno-chromatographic test (ICT) for malaria and kala-azar were reviewed. Histopathology reports were also reviewed by both pathologists and microbiologists.

Ethical consent

As it was a retrospective study, patient consent was not taken. However, approval from the ethical review committee of Dhaka Medical College Hospital was taken accordingly (ERC/DMC 12-07-2020). As one of our cases was HIV positive we maintained confidentiality regarding identifications and particulars.

## Results

**Figure 1:**
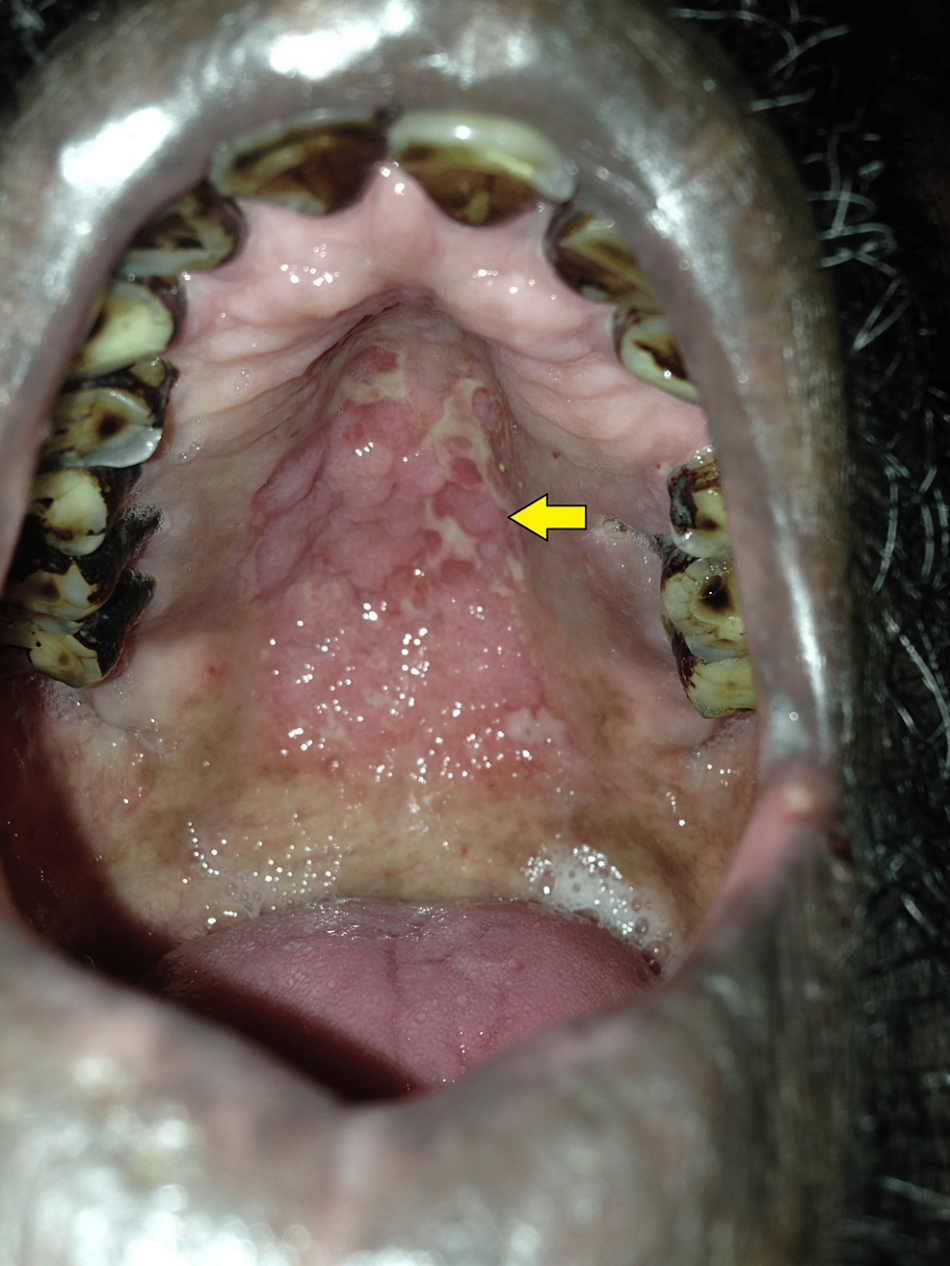
Oral ulceration of histoplasmosis on the hard palate (yellow arrow)

A total of nine patients were enrolled, male-to-female ratio was 3.5:1; the mean age was 47 (±13) years. Four of them used to smoke and one of them was an alcohol consumer. Three of them were poultry workers, two farmers, two truck drivers, and one sex worker by profession. The most common co-morbidities were diabetes 3 (33.3%) and hypertension 2 (22.2%). Fever 7 (77.7%), weight loss 6 (66.7%), hyperpigmentation 5 (55.5%), cough 4 (44.4%), oral ulceration 4 (44.4%) (Figure 1), anemia 4 (44.4%), lymphadenopathy 3 (33.3%) and low blood pressure with postural hypotension 3 (33.3%) were most common clinical presentations. Six (66.7%) of them had disseminated or systemic histoplasmosis. Seven (77.8%) out of nine patients were cured of histoplasmosis; however, one died before initiating antifungal medications and another one died due to a hypersensitivity reaction to liposomal amphotericin B (Table 1).

**Table 1:** Socio-demographic and clinical characteristics of patients infected with histoplasmosis (N = 9)

Trait	Frequency (%)
Age (Mean ± SD)	47 ± 13.2
Sex	
Male	7 (77.7)
Female	2 (22.3)
Smoker	4 (44.4)
Alcoholic	1 (11.1)
Occupation	
Farmer	2 (22.2)
Poultry worker	3 (33.3)
Businessman	1 (11.1)
Truck driver	2 (22.2)
Sex worker	1 (11.1)
Comorbidities	
Diabetes	3 (33.3)
Hypertension	2 (22.2)
Chronic kidney disease	1 (11.1)
Chronic liver disease	1 (11.1)
Asthma	1 (11.1)
Renal Transplant	1 (11.1)
Clinical Features	
Fever	7 (77.7)
Cough	4 (44.4)
Weight loss	6 (66.7)
Oral ulcer	4 (44.4)
Hyperpigmentation	5 (55.5)
Diarrhea	2 (22.2)
Hypotension	3 (33.3)
Anemia	4 (44.4)
Cervical lymphadenopathy	3 (33.3)
Hepatomegaly	2 (22.2)
Hepato-splenomegaly	1 (11.1)
Organ involvement	
Lungs	2 (22.2)
Adrenal gland	3 (33.3)
Liver/spleen	2 (22.2)
Type	
Localized	3 (33.3)
Disseminated	6 (66.7)
Outcome	
Cured	7 (77.8)
Death	2 (22.2)

Laboratory findings revealed low hemoglobin (median 9.8 g/dl), elevated ESR (median 39 mm), reduced absolute neutrophil count (median 3340/mm3), hyponatremia (median 127 mmol/l), elevated liver enzyme (median 57 IU/L) and hypoalbuminemia (median 3.3 g/dl). HIV infection was detected in 3 (33.3%) cases. Serological tests like hepatitis virus marker, ICT for malaria and kala-azar were negative. Cortisol values both in basal and 30 min after synthetic ACTH (adrenocorticotropic hormone) level were reduced (median 116.27 nmol/l and 217 nmol/l respectively) (Table 2).

**Table 2:** Laboratory profile of patients with histoplasmosis on admission Hb - Hemoglobin, ESR - Erythrocyte sedimentation rate, ALT - Alanine aminotransferase, AST - Aspartate aminotransferase, ACTH - Adrenocorticotropic hormone, AFB - Acid-fast bacillus, ICT- Immuno-chromatographic test, HBsAg- Hepatitis B virus surface antigen, HCV - Hepatitis C virus, HIV - Human immunodeficiency virus

Trait	Value (median)	Reference
Hb	9.8 (8.7-12.3)	11.5-15.5 g/dl
ESR	39 (21-59)	10-20 mm in 1^st^ hour
White blood cell count	5820 (5100-7880)	4000-11000 mm^3^
Absolute neutrophil count	3340 (1775-4290)	2000-7000 mm^3^
ALT	57 (41-82)	4-36 IU/L
AST	48 (35-71)	8-33 IU/L
Creatinine	1.10 (0.53-1.79)	0.5-1.3 g/dl
Albumin	3.3 (2.5-4.1)	3.5-4.5 g/dl
Bilirubin	0.75 (0. 3-1.7)	0.2-1.2 mg/dl
Random blood sugar	5.7 (4.1-12.9)	3.9-5.6 mmol / L
Na+	127 (119-147)	135-145 mmol / L
K+	4.2 (3.5-5.3)	3.5-5.5 mmol/L
Anti-HIV 1,2 antibody	3	
Serum cortisol (base) nmol/l	116.27 (89.4- 289.3)	101-690 nmol/l
Serum cortisol (30 min after ACTH)	217 (157-341)	≥ 690 – normal (nmol/l) 101-599 – partial insufficiency < 101 – complete insufficiency
Mantoux test	4 (2-11)	2-10 mm
Sputum for AFB stain and GeneXpert	negative	
ICT for malaria and kala-azar	negative	
HBsAg, anti-HCV	negative	
Biopsy from local ulcer	3	
Biopsy from lymph node	3	
Biopsy from the adrenal gland	2	
Broncho alveolar lavage with fungal stain	1	
Chest X-ray		
Miliary mottling	1	
Interstitial infiltration	1	
Cavitation	1	

**Figure 2:**
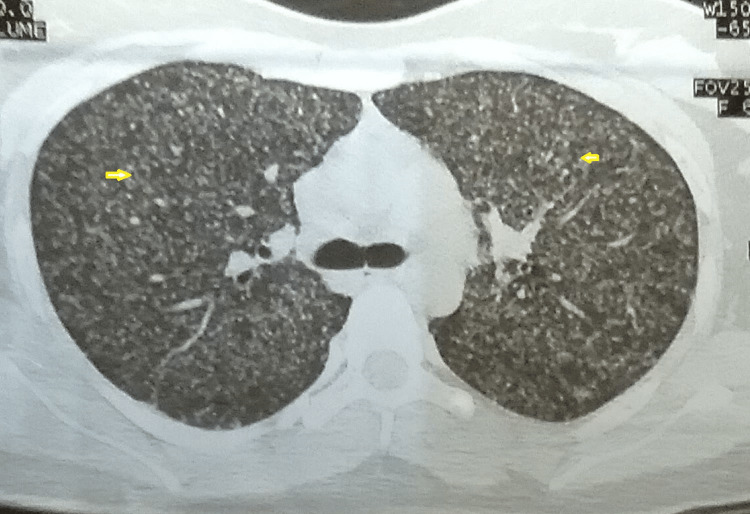
CT scan chest showing miliary mottling throughout both lungs (yellow arrow)

Chest X-rays in two pulmonary cases showed interstitial infiltration; band cavitation, and miliary mottling in the CT chest (Figure 2). 

**Figure 3:**
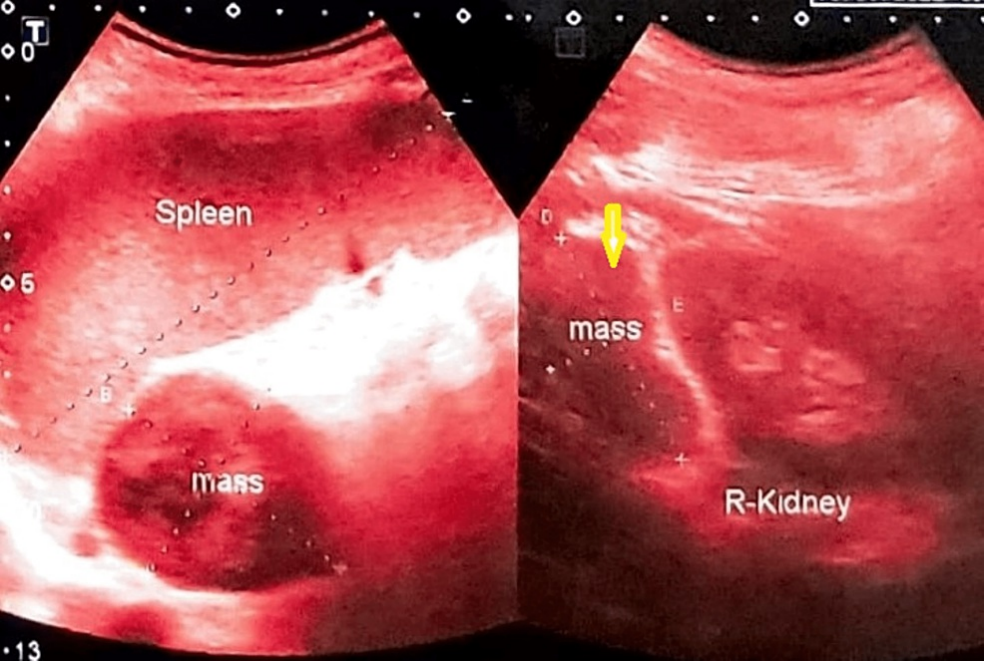
Systemic histoplasmosis involving the adrenal gland (yellow arrow)

Ultrasonograms of the whole abdomen showed an adrenal mass in three patients (Figure 3), hepatomegaly in two patients, and hepato-splenomegaly in one patient.

**Figure 4:**
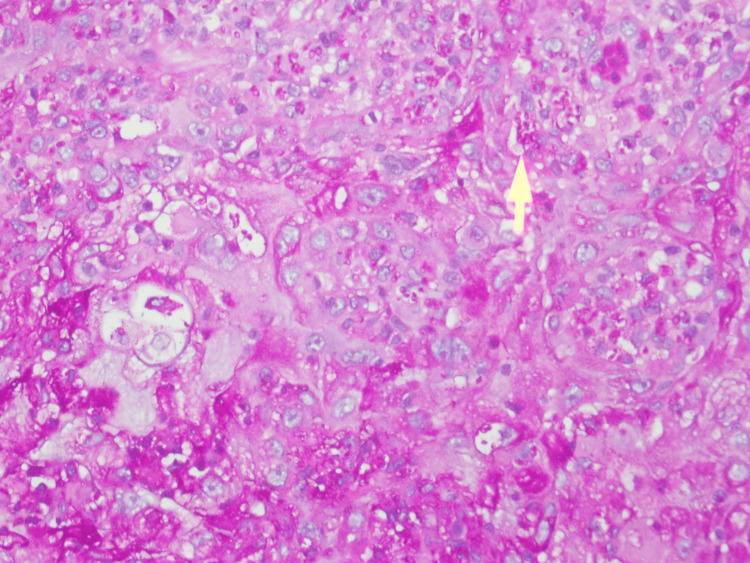
Histopathology with fungal stain revealed sheets of macrophages with numerous yeast cells of Histoplasma capsulatum (oral lesion) (yellow arrow) (periodic acid-Schiff stain, 40× magnification)

**Figure 5:**
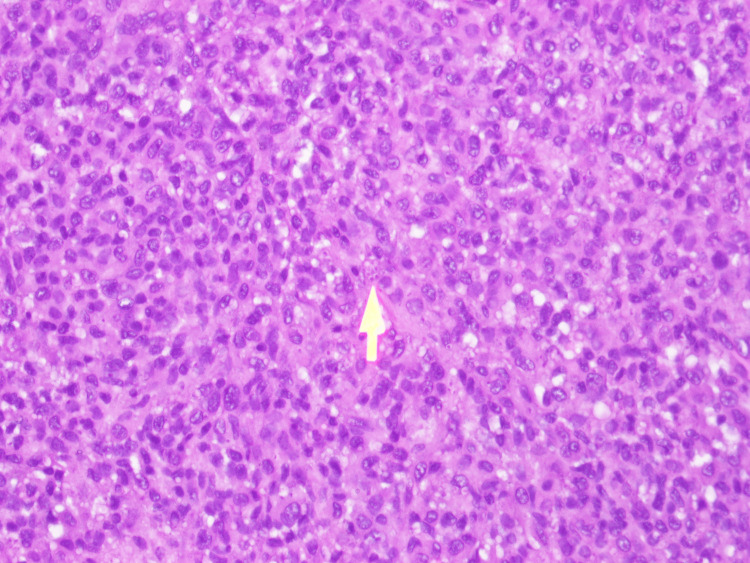
Histopathology with fungal stain revealed sheets of macrophages with numerous yeast cells of Histoplasma capsulatum (lymph node) (yellow arrow) (periodic acid-Schiff stain, 40× magnification)

Biopsy from the oral ulcer and the lymph node was done followed by histopathology with a fungal stain revealed sheets of macrophages with numerous yeast cells of *H. capsulatum* (Figure 4, 5).

## Discussion

Histoplasmosis is an opportunistic infection that occurs in immune-compromised persons but can occur in immune-competent hosts also [10]. It is endemic in the central, north, and south parts of America, however, it is rare in Bangladesh. Histoplasmosis has a predilection to the male gender; interestingly our patients were mostly male, farmers, or poultry workers by profession [11]. Fever, weight loss, oropharyngeal ulcer, lymphadenopathy, hepatosplenomegaly, and even bilateral adrenal enlargement were also common [1,9,12]. Similarly, most of our patients had clinical features like fever, oral ulceration, weight loss, cough, lymphadenopathy, hepato-splenomegaly, and bilateral adrenal mass. In addition, three of our patients presented with dizziness and hypotension and had partial adrenal insufficiency due to adrenal involvement. Wongprommek and Chayakulkeeree described that 3.5% of their patients suffered from hypotension [10]. It is worthwhile to mention four of our patients had anemia (microcytic hypochromic); among them, three were HIV patients who suffered frequent diarrhea and one had chronic kidney disease. So, it is important to find out the cause of anemia among histoplasmosis because histoplasmosis itself can cause anemia by bone marrow suppression [10,12].

An elevated liver enzyme, raised lactate dehydrogenase (LDH), and cytopenia are the established laboratory features of disseminated histoplasmosis [12,13]. We have observed more or less similar laboratory features among our patients who had concomitant HIV infection. Moreover, three of our patients had partial adrenal insufficiency evidenced by a positive short synacthen test. Among them, two were HIV positive and had diarrhea, and one had an associated upper respiratory tract infection that might precipitate adrenocortical insufficiency. Alam et al. and Afsana et al. from Bangladesh reported two different cases of adrenal insufficiency related to chronic disseminated histoplasmosis as a rare presentation [9,14].

Azoles are the treatment of choice for histoplasmosis in the management of a mild disease or as step-down therapy from amphotericin B in severe or disseminated disease [6]. Itraconazole is the Infectious Diseases Society of America guideline recommended choice for azole therapy based on prospective data. Out of six disseminated cases, four were cured, and two died. Patients who died had concomitant AIDS-defining illness, were not on highly active antiretroviral therapy (HAART) therapy, and were in septic shock; so, liposomal amphotericin B was not given. In another case, after giving a test dose of liposomal amphotericin B there was no reaction, however, during the second day of amphotericin B he suffered from a severe hypersensitivity reaction although methylprednisolone was given before it, liposomal amphotericin B was stopped immediately and intramuscular (IM) adrenaline was given thereafter. Eventually, he developed respiratory failure, and acute kidney injury and was sent to the intensive care unit (ICU) for mechanical ventilation and passed away 2 weeks later due to hospital-acquired pneumonia. Nath et al. reported two cases of severe hypersensitivity reactions to liposomal amphotericin B that were given to kala-azar patients [15].

The present study has a few limitations, like small sample size, retrospective study design, and lack of fungal culture, that is, the culture of histoplasmosis. In Bangladesh, there are great limitations in performing the culture of histoplasmosis. That is why we went for only a specific stain (periodic acid-Schiff stain) with characteristics of histopathological findings of histoplasmosis to confirm the diagnosis.

## Conclusions

For mild histoplasmosis, oral itraconazole is an effective antifungal medication. However, in moderate to severe form or disseminated histoplasmosis liposomal amphotericin B for at least two weeks followed by oral itraconazole for 12 weeks is still one of the preferable and most likely effective treatment options. Although the chance of hypersensitivity to liposomal amphotericin B is well known, caution should be taken to prevent and manage the hypersensitivity reaction appropriately.
